# BACE1 Processing of NRG1 Type III Produces a Myelin-Inducing Signal but Is Not Essential for the Stimulation of Myelination

**DOI:** 10.1002/glia.21255

**Published:** 2011-11-02

**Authors:** Viktorija Velanac, Tilmann Unterbarnscheidt, Wilko Hinrichs, Maike N Gummert, Tobias M Fischer, Moritz J Rossner, Amelia Trimarco, Veronica Brivio, Carla Taveggia, Michael Willem, Christian Haass, Wiebke Möbius, Klaus-Armin Nave, Markus H Schwab

**Affiliations:** 1 Department of Neurogenetics, Max-Planck-Institute of Experimental MedicineGoettingen, Germany; 2 Division of Neuroscience and INSPE, San Raffaele Scientific InstituteMilan, Italy; 3 DZNE-German Center for Neurodegenerative DiseasesMunich, Germany; 4 Biochemistry, Adolf-Butenandt-Institute, Ludwig-Maximilians-UniversityMunich, Germany

**Keywords:** PNS, Schwann cell, axon, growth factor

## Abstract

Myelin sheath thickness is precisely adjusted to axon caliber, and in the peripheral nervous system, neuregulin 1 (NRG1) type III is a key regulator of this process. It has been proposed that the protease BACE1 activates NRG1 dependent myelination. Here, we characterize the predicted product of BACE1-mediated NRG1 type III processing in transgenic mice. Neuronal overexpression of a NRG1 type III-variant, designed to mimic prior cleavage in the juxtamembrane stalk region, induces hypermyelination *in vivo* and is sufficient to restore myelination of NRG1 type III-deficient neurons. This observation implies that the NRG1 cytoplasmic domain is dispensable and that processed NRG1 type III is sufficient for all steps of myelination. Surprisingly, transgenic neuronal overexpression of full-length NRG1 type III promotes hypermyelination also in BACE1 null mutant mice. Moreover, NRG1 processing is impaired but not abolished in BACE1 null mutants. Thus, BACE1 is not essential for the activation of NRG1 type III to promote myelination. Taken together, these findings suggest that multiple neuronal proteases collectively regulate NRG1 processing. © 2011 Wiley Periodicals, Inc.

## INTRODUCTION

Matching myelin sheath thickness to axon caliber is critically important for optimal nerve impulse conduction and requires reciprocal signaling between axons and glial cells. A key regulator of peripheral myelination is the growth factor neuregulin (NRG) 1 type III (Birchmeier and Nave,^2008^; Nave and Salzer,^2006^). This axonal protein is not only required for embryonic development of the Schwann cell lineage (Wolpowitz et al.,^2000^) but also NRG1 type III regulates the ensheathment fate of axons (Taveggia et al.,^2005^) and myelin sheath thickness (Michailov et al.,^2004^).

NRG1 type III is a member of a large family of growth and differentiation factors encoded by the single *Nrg1* gene (Falls,^2003^; Mei and Xiong,^2008^). All NRG1 isoforms share an epidermal growth factor (EGF)-like extracellular domain and activate receptor tyrosine kinases of the ErbB family (Mei and Xiong,^2008^).

NRG1 type III is characterized by a cysteine-rich domain (CRD), which serves as a second N-terminal transmembrane domain (Cabedo et al.,^2002^; Ho et al.,^1995^; Schroering and Carey,^1998^). Thus, its membrane topology suggests a tight association of the signaling domain with the cell membrane (Wang et al.,^2001^). The identification of truncated NRG1 isoforms in tissue extracts has suggested that proteolytic processing of full-length NRG1 isoforms regulates its functions (Jessell et al.,^1979^; Lemke and Brockes,^1984^). When analyzed in cultured cells, several proteases, including ADAM19 (β-meltrin), TACE (ADAM17), and BACE1 (β-site amyloid precursor protein–cleaving enzyme1), can cleave the membrane-anchored forms of NRG1 type I and II. By acting as “sheddases,” they release the EGF-like domain (Horiuchi et al.,^2005^; Hu et al.,^2008^; Montero et al.,^2000^; Shirakabe et al.,^2001^; Willem et al.,^2006^).

For ADAM19-deficient mice, a delay of remyelination after nerve crush has been documented, but the developmental time course of myelination appears normal (Wakatsuki et al.,^2009^). TACE cleaves recombinant NRG1 within the EGF-like domain and reduces active NRG1 type III on the axonal surface. Thus, inactivation of TACE in spinal motor neurons causes hypermyelination *in vivo* (La Marca et al.,^2011^). In contrast, BACE1-deficient mice are hypomyelinated in the PNS (Willem et al.,^2006^) and accumulate full-length NRG1 isoform(s) in the brain. Finally, mouse mutants lacking the zinc peptidase nardilysin, an upstream activator of both TACE and BACE1, exhibit also peripheral hypomyelination (Ohno et al.,^2009^), with a similar accumulation of full-length NRG1 in the brain. Thus, processing of NRG1 type III in peripheral nerves could be part of an activation step that is critical for axon-glia signaling. Collectively, these data suggest a working model in which BACE1 activates axonal NRG1 type III, which subsequently stimulates Schwann cells to make myelin.

In this study, we explored NRG1 type III processing and the functional interaction between NRG1 type III and BACE1 during myelination *in vivo*. NRG1 processing is reduced but not abolished in the absence of BACE1, and neuronal overexpression of full-length NRG1 type III promotes hypermyelination also in BACE1 null mutant mice. This suggests a revised model in which multiple proteases collectively regulate NRG1 type III during myelination *in vivo*.

## MATERIALS AND METHODS

### Transgenic Constructs

HA-NRG1^FL^ was generated by PCR from murine NRG1 type IIIβ1a cDNA (kindly provided by C. Lai), which added two consecutive hemagglutinin (HA) peptide tags to the N-terminus. HA-NRG1^GIEF^ was generated by PCR from HA-NRG1^FL^ cDNA by introducing a stop codon after the “GIEF” coding sequence. Primer sequences are available upon request. PCR products were verified by DNA sequencing. Both HA-NRG1^FL^ and HA-NRG1^GIEF^ cDNAs were subcloned into the XhoI site of the Thy1.2 expression cassette (Caroni,^1997^).

### Mouse Lines

To generate HA-NRG1^FL^ and HA-NRG1^GIEF^ transgenic mouse lines, the corresponding Thy1.2 expression cassettes were excised from the plasmid backbone by PvuI/NotI digest and injected into fertilized FVB (HA-NRG1^FL^) or C57BL/6 (HA-NRG1^GIEF^) mouse oocytes. One founder each was used to establish HA-NRG1^FL^ and HA-NRG1^GIEF^ mouse lines. Transgenic line HA-NRG1^FL^ was backcrossed to C57BL/6 for more than 10 generations. BACE1 mutants (Cai et al.,^2001^) were purchased from Jackson Laboratory. Genotyping primer sequences are available upon request. All mouse experiments were performed in compliance with animal policies approved by the State of Lower Saxony.

### RNA Expression Analysis

RNA isolation was performed using the RNeasy Mini Prep kit (Qiagen). cDNA was synthesized from 500 ng RNA using random nonamers and Superscript III Reverse Transcriptase (Invitrogen). RT-PCR to verify transgene expression was performed using primers: (forward) 5′-GGTGCAG CAACTGGAGG CGTTG-3′ and (reverse) 5′-GTCCACAAATACCCACTTTAGGCCAGC-3′. Tubulin was amplified with primers (forward) 5′-GATATCGCTGCGCTGGTCGTC-3′ and (reverse) 5′-CATGGCTGGGGTGTTGAAGGTC-3′.

### Cell Culture and Immunoblotting

HEK293T cells were grown on poly-L-lysine (PLL) coated culture dishes and transfected using Lipofectamine 2000 (Invitrogen) at 70% confluency. The following plasmids were used for transfections: pCMV2-HA-NRG1^FL^, pCMV2-HA-NRG1^GIEF^, and pcDNA3.1-BACE1 (Westmeyer et al.,^2004^). Protease inhibitor β-secretase inhibitor IV (2 μM; Calbiochem) was added 2 h after transfection. Protein lysates were prepared 24 h after transfection with RIPA buffer supplemented with protease inhibitors (Complete mini, Roche). A total of 10 μg of protein lysates was used for Western blotting.

### Split-TEV Assay

Split-TEV assays for the analysis of protein–protein interactions at the cell surface were performed essentially as described (Wehr et al.,^2006^). Briefly, NRG1 constructs (pCMV2-HA-NRG1^FL^, pCMV2-HA-NRG1^GIEF^) were transfected into PC12 cells by lipofection (Lipofectamin 2000; Invitrogen). NRG1-transfected cells were added to PC12 cells expressing ErbB2 and ErbB3 receptor variants fused to inactive N- (ErbB2N-TEV) and C-terminal (ErbB3-C-TEV) fragments of the tobacco etch virus (TEV) protease. ErbB2N-TEV is additionally fused to a transcription factor (GV) through the TEV protease cleavable peptide sequence tevS (ErbB2N-TEV-tevS-GV). NRG1-mediated ErbB receptor dimerization brings the two inactive TEV protease fragments in close proximity, which reconstitutes protease activity, and activates luciferase reporter gene through GV release. Luciferase activity was measured using the Mithras LB 940 Plate Reader (Bertholde). Results were displayed as relative luminescence units (RLUs). A one-sided Student's *t*-test was used for statistical analysis.

### Dissociated Dorsal Root Ganglia Explants

Dorsal root ganglia (DRG) were dissected from C57Bl/6 mouse embryos at E13 as described (Saher et al.,^2009^). Briefly, dissected DRG were triturated in 0.25% trypsin at 37°C. Cells were plated onto collagen-coated 18-mm coverslips and maintained in MEM+l-glutamine media supplemented with 10% FBS and 50 ng/μl 2.5S nerve growth factor (NGF) (Alomone Labs). Myelination was induced after 1 week by supplementing medium with 50 μg/ml ascorbic acid (Sigma). Myelinating growth medium was changed every 2nd day. Treatment with 2 μM β-secretase inhibitor IV and 1 μM ADAM's protease inhibitor GM6001 (Calbiochem) were initiated 2 days before induction of myelination. Protease inhibitors were added freshly in myelinating growth medium. Control coverslips were treated with with dimethyl-sulfoxide (DMSO). Cultures were analyzed 7 or 14 days after induction of myelination by immunostaining for MBP and tubulin. Briefly, cells were fixed for 10 min in 4% PFA in phosphate buffer (PB), permeabilized 5 min at −20°C with ice-cold methanol, and blocked for 1 h in 3% BSA in PBS. Primary antibodies were incubated overnight in blocking solution at RT. Secondary antibodies and DAPI (1:2000) in blocking solution were applied for 1 h at RT. Antibodies: rabbit anti-MBP (1:200; DAKO), mouse Tuj1 (1:500; Covance), anti-rabbit IgG-cy2 (1:100; Dianaova), and anti-mouse IgG-cy3 (1:1000; Dianova). Quantified numbers of myelin segments and nuclei were statistically analyzed using one-sided Student's *t*-test.

### DRG-Schwann Cell Co-cultures

DRG neurons were prepared from E14 wildtype or NRG1 type III-specific (CRD−/−) null mutant embryos (Wolpowitz et al.,^2000^). Selection of sensory neurons by cycles of 5-fluorodeoxyuridine (FDUR) treatment, preparation, and addition of rat Schwann cells to the established DRG neuron culture was essentially as described (Taveggia et al.,^2005^). Myelination was initiated by addition of ascorbic acid to media (50 μg/mL) 2 days after addition of rat Schwann cells. Myelination was assessed by immunostaining for MBP-positive myelin segments 1 or 2 weeks after myelination induction. Antibodies: mouse anti-MBP (1:250; Sternberger Monoclonal) and chicken anti-NF (1:2000; Covance). For Western blotting, co-cultures lysed in lysis buffer (95 mM NaCl, 25 mM Tris pH 7.5, 10 mM EDTA, 2% SDS, 1 mM NaF, 1 mM Na_3_VO_4_, 1% protease inhibitor), 12 days after myelination induction. Membranes were probed with mouse anti-MBP (1:4000; Sternberger Monoclonal) and rabbit anti-actin (1:4000; Sigma).

### Generation of Lentiviruses and Infection of DRG Neurons

HA-NRG1^FL^ and HA-NRG1^GIEF^ cDNAs were subcloned into pLenti6/V5-DEST for lentiviral expression by gateway cloning. Primer sequences are available upon request. Lentiviruses expressing HA-NRG1^FL^ and HA-NRG1^GIEF^ were generated using the ViraPower Lentiviral Expression System (Invitrogen) as described (Taveggia et al.,^2005^). For viral infection, cultures were incubated with viral supernatants in DRG culture media for 16 h immediately after dissection. Viral expression was confirmed by anti-HA staining 11 days postinfection using rabbit anti-HA antibody (1:100; Sigma).

### Protein Analysis

Tissues were homogenized in RIPA buffer supplemented with protease inhibitors (Complete mini, Roche). Protein concentrations were determined using the Bio-Rad DC protein assay. Typically, 30–40 μg of protein lysates was separated by SDS-PAGE electrophoresis and transferred onto PVDF membranes (Amersham-Hybond P). Membranes were blocked in TBST (TBS/0.05%Tween) with 5% milk and incubated over night with primary antibody at 4°C. After washing in TBST, membranes were incubated with appropriate HRP-conjugated secondary antibodies for 1 h at RT and developed using the ECL plus detection kit (Perkin Elmer Life Sciences). The following primary antibodies were used: rat anti-HA tag antibody (1:1000; Roche), rabbit anti-carboxy terminus of neuregulin1 (1:500, Santa Cruz sc-348), rat anti-GIEF (1:50; clone 4F10; Michael Willem unpublished), rabbit anti-Necl1 (1:1000; gift from Elior Peles), and mouse anti-α-tubulin (1:2000; Sigma). HRP-conjugated secondary antibodies (Dianova) were used at 1:5000 dilution. Western blot quantification was performed using ImageJ software. Values were normalized to tubulin. A two-sided Student's *t*-test was used for statistical analysis.

### Histology and Immunohistochemistry

Mice were anesthetized with avertin (0.2 mL/10 g) and perfused with 4% PFA in 0.1 M PB. Dissected tissue was postfixed overnight at 4°C and embedded in paraplast. 5 μm thick microtome sections was subjected to histological analysis. For fluorescent immunostaining, sections were blocked with 20% goat serum in PBS/BSA, incubated with primary antibodies overnight at 4°C, followed by incubation with the corresponding cy2 (1:100; Dianova) or cy3 (1:1000; Dianova) coupled secondary antibodies for 1 h at RT. The following primary antibodies were used: mouse anti-HA tag (1:250; Covance), rabbit anti-carboxy terminus of neuregulin1 (1:100; Santa Cruz sc-348), rabbit anti-neurofilament 200 (1:200; Sigma), rabbit anti-peripherin (1:500; Chemicon), mouse anti-class III β-tubulin (1:250; Covance), mouse anti-NeuN (1:100; Chemicon), rabbit anti-parvalbumin (1:200, Swant), rabbit anti-Olig2 (1:100; gift from John Alberta), and rabbit anti-Necl1 (1:1000; gift from Elior Peles). Nuclei were counterstained with DAPI (1:2000). Images of fluorescently labeled tissue were obtained with a SP2-AOBS Leica confocal microscope and processed using Photoshop CS3 and ImageJ software.

### Electron Microscopy and Morphological Analysis

Mice were transcardially perfused with Karlsson and Schulz fixative (2.5% glutaraldehyde and 4% PFA in PB). Dissected sciatic nerves were embedded in epoxy resin. Semithin sciatic nerve cross sections (0.5 μm) were cut on a microtome (Ultracut S, Leica) with a diamond knife (Diatome Histo 45°) and stained for light microscopy with Methylenblue-Azur II for 1 min at 60°C. Ultrathin sections (50 nm) for electron microscopy were cut with a diamond knife (Diatome Ultra 35°) placed on a copper slot grid (2 mm–1 mm, AGAR) coated with Formvar polyvinyl and contrasted. For *g*-ratio measurement and quantification of axon and Schwann cell numbers images were obtained with the DMRXA (Leica) microscope with a Kappa DX20 H-FW digital camera. G-ratios of at least 100 myelinated axons per mouse were measured using the ImageJ g-ratio plug-in. For ultrastructural analysis, sections were imaged using a LEO EM912 Omega electron microscope (Zeiss) with an on-axis 2048 × 2048-CCD camera (Proscan). All images were processed using Photoshop CS3 and ImageJ software. For statistical analysis of the quantifications a one-sided Student's *t*-test was used.

### Immuno-EM

For immunoelectron microscopy, mice were transcardially perfused with 0.2% glutaraldehyde and 4% PFA in 0.1 M PB. Small pieces of the cortex were incubated in 2.3 M sucrose in 0.1 M PB overnight. Tissue blocks were mounted onto aluminum pins for ultramicrotomy and frozen in liquid nitrogen. Ultrathin cryosections were cut with a diamond knife (Diatome) at −110°C using a cryo-ultramicrotome UC6 (Leica) and picked up in a 1:1 mixture of 2% methylcellulose and 2.3 M sucrose. Thawed sections were immunolabeled as described (Peters and Pierson,^2008^). For double-immunolabeling, sections were incubated with a polyclonal antibody directed against PSD95 (Fukaya and Watanabe,^2000^), followed by protein A gold conjugate (15 nm) purchased from the Cell Microscopy Center, Department of Cell Biology, University Medical Center Utrecht, The Netherlands. Next, a monoclonal mouse anti-HA antibody (Covance) was used as primary antibody followed by incubation with a secondary rabbit antibody directed against mouse IgG (Rockland) and a protein A gold conjugate (10 nm).

## RESULTS

### Proteolytic Cleavage of NRG1 type III in the Juxtamembrane “Stalk” Region Activates a Myelination Signal

**Fig. 1:**
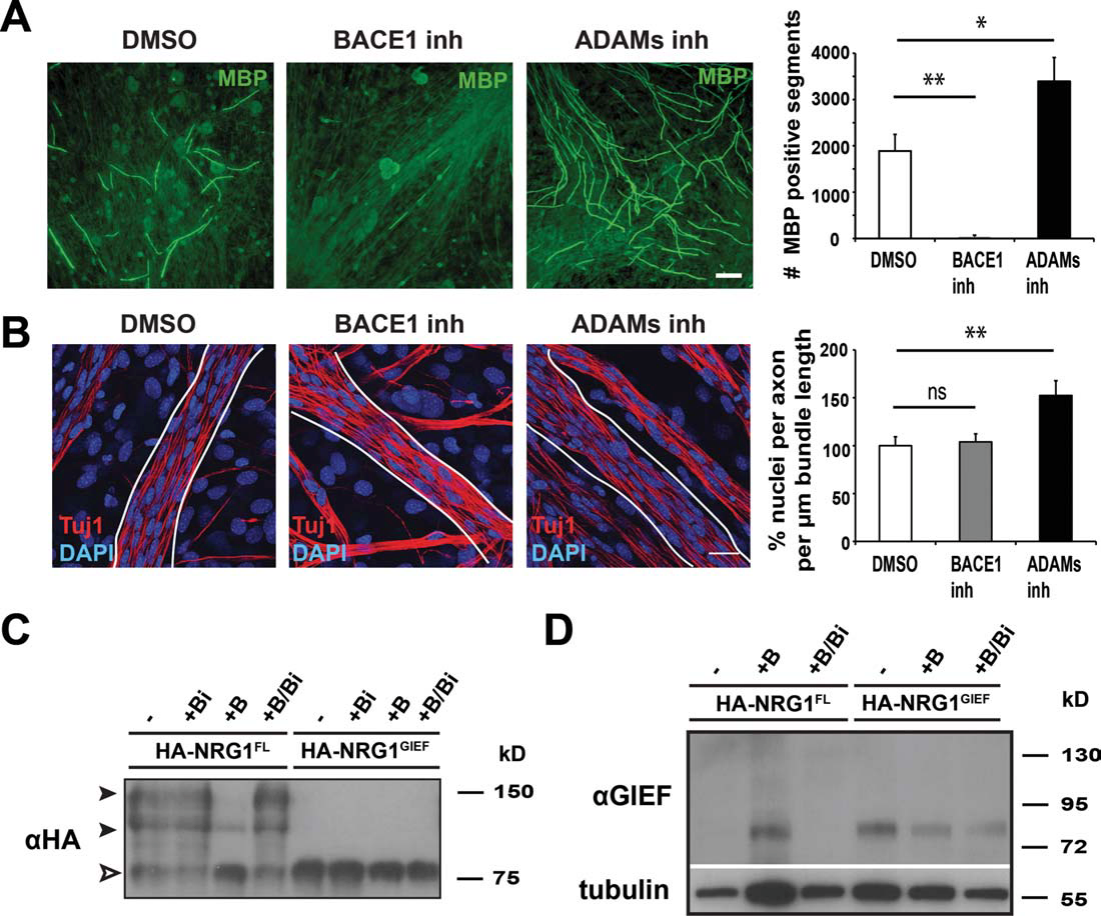
Proteases serve as positive and negative regulators of myelination. (**A**) Pharmacological BACE1 inhibition blocks myelination in DRG dissociated explant cultures, whereas inhibition of ADAMs promotes myelination. Fluorescent imunostaining for MBP (green). Numbers of MBP-positive segments (mean ± SEM) after 14 days *in vitro* (DMSO, ADAMs inh *n* = 3 cultures; BACE1 inh *n* = 2 cultures; 2 coverslips/treatment; *P < 0.05, **P < 0.01). Scale bar, 200 μm. (**B**) Axonal association of Schwann cells is not impaired upon BACE1 or ADAMs inhibition in DRG neuron dissociated explant cultures. Immunostaining for axons (Tuj1; red) and counterstaining for nuclei (DAPI; blue) after 9 d of protease inhibitor treatment. Representative axon bundles used for quantification are delineated. Numbers of axon-associated Schwann cells (per bundle length in μm) are displayed as percentage ± SEM relative to DMSO treated controls (*n* = 2 cultures; 3 coverslips/condition; 3 bundles/coverslip; *P* = 0.38, **P < 0.01). Scale bar, 20 μm. (**C**) Cleavage of full-length (HA-NRG1^FL^), but not “processed” NRG1 type III (HA-NRG1^GIEF^) by BACE1. Western blot of transfected HEK293T cell lysates probed with an anti-HA antibody. Expression of BACE1 (+B) strongly reduced the level of full-length HA-NRG1^FL^ (black arrowheads) and resulted in the accumulation of a ∼75 kD N-terminal processing product (white arrowhead), which corresponds in size to HA-NRG1^GIEF^. HA-NRG1^FL^ processing by BACE1 was blocked by BACE1 inhibitor treatment (+B/Bi). Expression of BACE1 had no visible impact on HA-NRG1^GIEF^ levels. Note that “base line” processing of HA-NRG1^FL^ (+Bi) was not affected by BACE1 treatment. (**D**) BACE1 cleaves HA-NRG1^FL^ in the juxtamembrane “stalk” region. Western blot of transfected HEK293T cell lysates probed with an antibody against the C-terminal peptide sequence “GIEF”. Note that the C-terminal in ‘GIEF’ epitope only accumulates in HA-NRG1^FL^ lysates when BACE1 was coexpressed (+B). HA-NRG1^GIEF^ lysates served as a positive control. The membrane was reprobed for tubulin as a loading control. [Color figure can be viewed in the online issue, which is available at wileyonlinelibrary.com.]

Several proteases have been implicated in NRG1 processing (Hu et al.,^2008^; La Marca et al.,^2011^; Montero et al.,^2000^; Wakatsuki et al.,^2009^; Werner et al.,^2008^; Willem et al.,^2006^). To screen for proteases that promote myelination, we treated myelinating DRG cultures (Saher et al.,^2009^) with protease inhibitors and quantified the number of MBP-positive segments 2 weeks after induction of myelination (Fig. 1A). Application of the BACE1 inhibitor “β-secretase inhibitor IV” (2 μM) almost completely blocked myelination compared with control cultures (inhibitor 36.25 ± 33.31; control 1889.83 ± 356.36 *P* < 0.01) (Fig. 1A), without affecting the number of axon-associated Schwann cells (inhibitor 103.97 ± 8.33; control 100 ± 9.22; *P* = 0.38) (Fig. 1B). However, when we prepared co-cultures from BACE1 deficient mice, myelination was impaired but not completely blocked (Supp. Info. Fig. 1). This supports a role of BACE1 in myelination (Hu et al.,^2006^; Willem et al.,^2006^) but also indicated the activity of additional proteases that might compensate for BACE1. Treatment of co-cultures with the ADAMs inhibitor GM6001 (1 μM), which preferentially targets TACE and several matrix metalloproteases (MMPs 1, 2, 8, 9), increased the number of MBP-positive segments 1.8-fold when compared with DMSO-treated control cultures (inhibitor 3393.5 ± 512.37; control 1889.83 ± 356.36; *P* < 0.05) (Fig. 1A) and moderately increased the number of axon-associated Schwann cells (inhibitor 152.5 ± 15.48; control 100 ± 9.22; *P* < 0.01). Thus, processing by ADAMs proteases such as TACE inhibits myelination, in line with recent findings (La Marca et al.,^2011^).

**Fig. 2:**
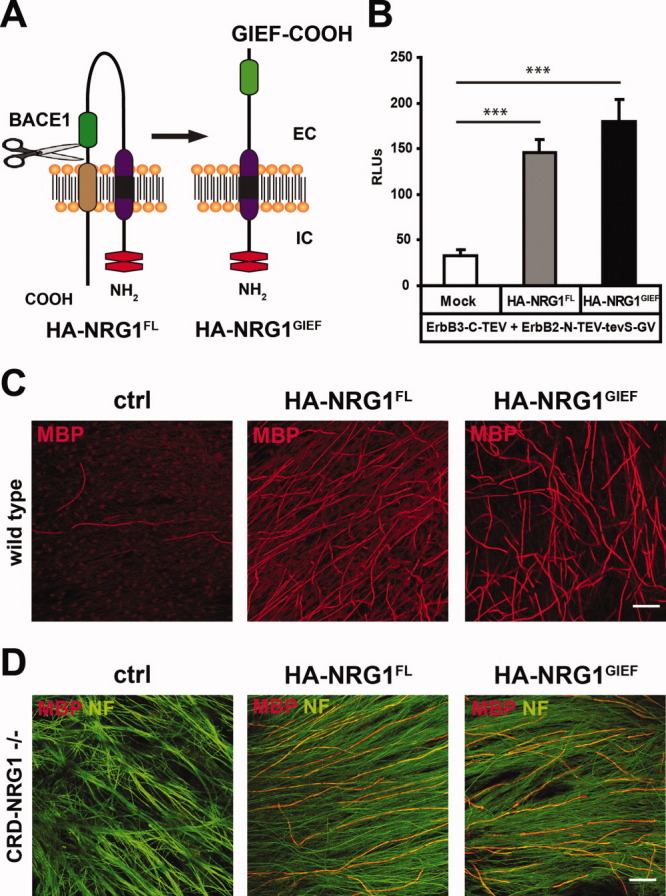
BACE1 cleavage in the “stalk” region produces a myelination-inducing NRG1 type III variant. (**A**) Domain structure of HA-NRG1^FL^ and HA-NRG1^GIEF^. BACE1 cleavage of HA-NRG1^FL^ is indicated by scissors. Protein domains: EGF-like domain (green), cystein-rich domain (purple/black), transmembrane domain (brown), HA epitope tags (red). EC: extracellular; IC: intracellular. (**B**) HA-NRG1^FL^ and HA-NRG1^GIEF^ induce heterodimerization of ErbB2 and ErbB3 receptors in a two-cell split-TEV assay as measured by a ∼5 fold increase in luciferase reporter activity compared with controls. Results are displayed as relative luminescence units (RLUs) ±SD (*n* = 6; ***P < 0.001). (**C**) HA-NRG1^FL^ and HA-NRG1^GIEF^ stimulate myelination in DRG neuron-Schwann cell co-cultures. Immunostaining for MBP (red) demonstrates a dramatic increase in the number of myelin segments upon lentiviral expression of HA-NRG1^FL^ or HA-NRG1^GIEF^ in sensory neurons compared with noninfected control cultures (ctrl) 7 days after myelination induction. Scale bar, 50 μm. (**D**) Sensory neurons prepared from NRG1 type III null mutants (CRD-NRG1−/−) are not myelinated 14 days after induction of myelination. Myelination is restored in NRG1 type III-deficient axons after lentiviral overexpression of HA-NRG1^FL^ or HA-NRG1^GIEF^ as demonstrated by immunostaining for MBP (red). Neurofilament (NF) staining (green) reveals the normal maintenance of sensory axons in mutant cultures. Scale bar, 50 μm. [Color figure can be viewed in the online issue, which is available at wileyonlinelibrary.com.]

Next, we examined the consequences of protease inhibition on the processing of an N-terminally HA-tagged NRG1 type III variant expressed in HEK293T cells. Western blotting of protein lysates from cells expressing full-length NRG1 type III (HA-NRG1^FL^; Fig. 2A), using an anti-HA antibody, revealed three major bands (Fig. 1C). The two upper bands most likely represent differentially glycosylated “immature” (∼110 kD) and “mature” forms (∼140 kD) of the full-length NRG1 type III isoform. The protein with lower molecular weight (∼75 kD) was compatible with a cleavage product of full-length NRG1 type III, when processed in the juxtamembrane stalk region. This “base line” processing of NRG1 type III was unaltered in the presence of BACE1 inhibitor (Fig. 1C). However, overexpression of BACE1 dramatically diminished full-length NRG1 type III and resulted in the accumulation of processed NRG1 type III, which was fully blocked by the addition of BACE1 inhibitor (Fig. 1C). This suggests that proteases other than BACE1 can process NRG1 type III in HEK293T cells.

*In vitro*, BACE1 can cleave a polypeptide that models the stalk region of NRG1β1, thereby producing a peptide fragment, in which the C-terminal amino acid sequence reads “Gly-Ile-Glu-Phe-COOH” (Hu et al.,^2008^). We generated the corresponding “GIEF” variant (termed HA-NRG1^GIEF^) that was in addition N-terminally tagged with the HA epitope, mimicking NRG1 type IIIβ1a after BACE1 cleavage (Fig. 2A). Expression of HA-NRG1^GIEF^ in HEK293T cells produced one major band co-migrating with processed NRG1 type III (Fig. 1C). Co-expression of BACE1 (Fig. 1C) did not cause visible processing of HA-NRG1^GIEF^.

To determine whether BACE1 cleavage of full-length NRG1 type III produces the GIEF C-terminus, we used an antibody directed against the C-terminal “GIEF” epitope (Willem et al., unpublished). We clearly detected this epitope in HA-NRG1^GIEF^ transfected cell lysates, independent of BACE1 activity (Fig. 1D). In contrast, the GIEF epitope accumulated in HA-NRG1^FL^ lysates only when BACE1 was co-expressed (Fig. 1D). This demonstrates that BACE1 cleavage of NRG1 type III can occur at the GIEF site; however, there is also processing of NRG1 type III in HEK293T cells (by other proteases) that produces distinct C-termini.

We next assessed the capacity of HA-NRG1^GIEF^ to activate ErbB receptors in a two-cell split-TEV assay, which monitors cell surface expression and interaction of NRG1 ligands with their cognate ErbB receptors in co-cultures of two separately transfected cell populations (Wehr et al.,^2006^). Similar to HA-NRG1^FL^ (145.83 ± 13.83 RLUs; *P* < 0.001), expression of HA-NRG1^GIEF^ (180.08 ± 23.97 RLUs; *P* < 0.001) significantly induced the dimerization of ErbB2 and ErbB3 receptors when compared with mock transfected cells lacking the NRG1 ligand (33.11 ± 6.36 RLUs) (Fig. 2B). Thus, HA-NRG1^GIEF^ serves as a ligand for ErbB3 and activates ErbB2/ErbB3 heterodimers.

These data demonstrate a stimulatory role of BACE1 during myelination, which most likely involves activation of full-length NRG1 type III through proteolytic cleavage in the stalk region. In contrast, TACE (La Marca et al.,^2011^) and other ADAMs proteases may serve as negative regulators of peripheral myelination. Therefore, we focused our analysis on the functional consequences of the interaction between NRG1 type III and BACE1.

In DRG-Schwann cell co-culture, lentiviral expression of HA-NRG1^GIEF^ in sensory neurons dramatically increased the number of MBP-positive myelin segments, similar to HA-NRG1^FL^, when compared with noninfected controls (Fig. 2C). However, the large number of myelinated segments impeded reliable quantification. Sensory neurons from NRG1 type III null mutants (CRD-NRG1−/−) are not myelinated when wildtype Schwann cells are added (Fig. 2D**),** as reported previously (Taveggia et al.,^2005^). Lentiviral expression of HA-NRG1^GIEF^ in CRD-NRG1−/− sensory neurons fully restored myelination, similar to the myelination rescue by HA-NRG1^FL^ (Fig. 2D).

These data collectively demonstrate that proteolytic cleavage of NRG1 type III by BACE1 produces a transmembrane protein with type II topology that stimulates Schwann cell myelination in culture. In contrast, the C-terminal domain of NRG1 and the cytoplasmic tail are not required to induce myelination.

### Regulated Axonal Transport of NRG1 Type III *In Vivo*

**Fig. 3:**
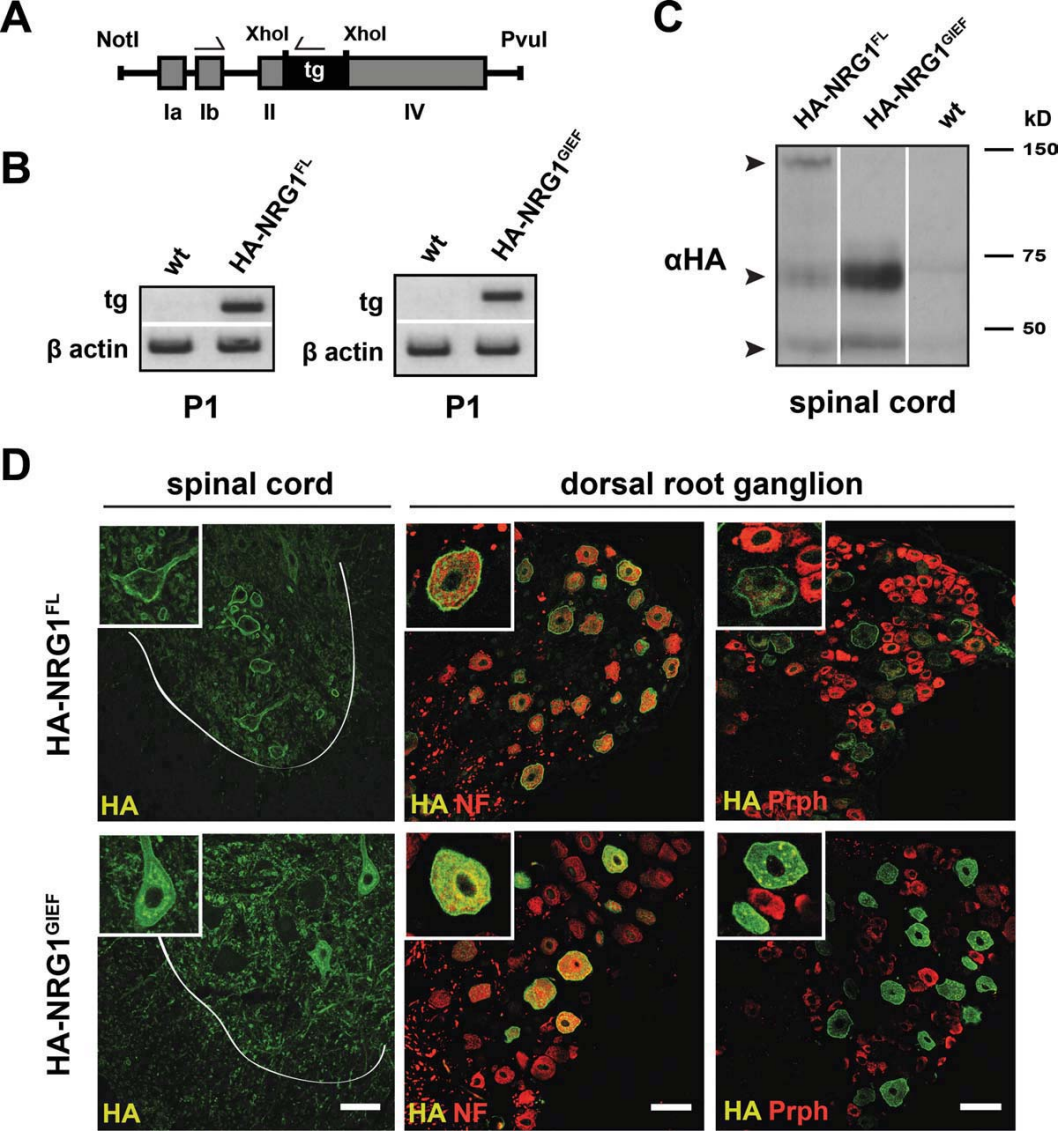
Neuronal overexpression of epitope-tagged NRG1 type III-variants in transgenic mice. (**A**) Schematic drawing of the Thy1.2 cassette used for neuronal expression of HA-NRG1^FL^ and HA-NRG1^GIEF^ cDNAs (tg). XhoI, restriction site for subcloning; NotI/PvuI, restriction sites for cassette release; exons Ia, Ib, II, IV, and locations of RT-PCR primers used in (**B**) are indicated. (B) HA-NRG1^FL^ and HA-NRG1^GIEF^ transgenes are expressed in spinal cord at the onset of peripheral myelination. Transgene-specific RT-PCR of spinal cord cDNAs from transgenic and wildtype (wt) mice at P1. Amplification of β-actin was used as a positive control. (**C**) HA-NRG1^FL^ and HA-NRG1^GIEF^ proteins are expressed in the spinal cord of transgenic mice. Western blot of spinal cord protein lysates (40 μg) at 2 months of age probed with an anti-HA antibody. Arrowheads mark full-length (∼ 140 kD) and processed HA-NRG1 type III (∼ 70 kD) as well as a lower molecular weight protein band (∼ 45 kD), which most likely results from further NRG1 type III processing. (**D**) Spinal cord motor neurons and sensory neurons express HA-NRG1^FL^ and HA-NRG1^GIEF^ proteins at their plasma membrane. Confocal images of spinal cord and DRG cross sections after immunostaining for the HA-epitope, neurofilament 200 (NF), and peripherin (Prph). White line marks the border between gray and white matter. Scale bars, 50 μm. [Color figure can be viewed in the online issue, which is available at wileyonlinelibrary.com.]

**Fig. 4:**
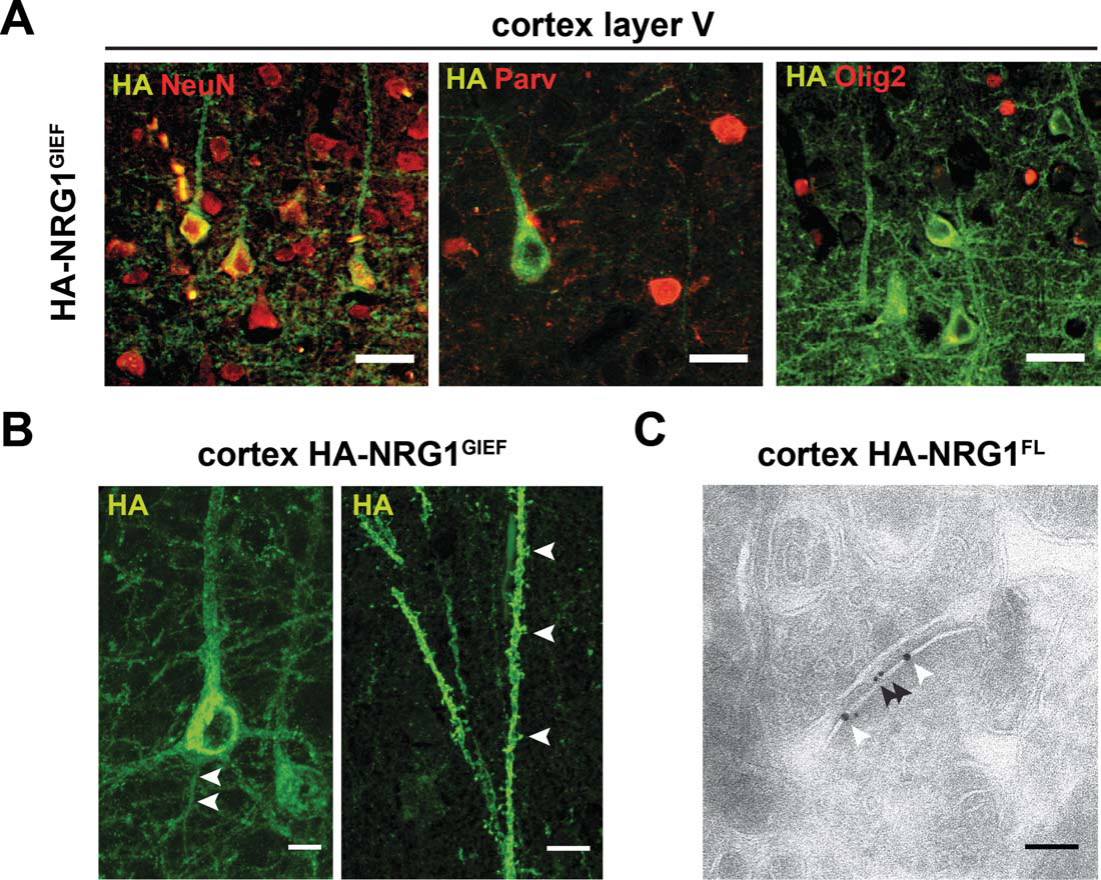
Expression of the epitope-tagged NRG1 type III-variants in the brains of transgenic mice. (**A**) HA-NRG1^GIEF^ expression in the cortex exclusively localizes to neurons. Confocal images of cortical layer V immunostained for the HA-epitope and cell type-specific markers. HA-NRG1^GIEF^ accumulates in a subset of NeuN-positive cortical neurons but is absent from parvalbumin (Parv)-expressing interneurons and Olig2-positive oligodendrocytes. Scale bars, 25 μm. (**B**) Confocal image of a HA epitope-positive neocortical projection neuron from a HA-NRG1^GIEF^ transgenic mouse at 4 months of age. HA-NRG1^GIEF^ is expressed in the soma, axon (arrowheads), and proximal dendrites (left panel), as well as dendritic spines (right panel; arrowheads). Scale bars, 10 μm. (**C**) Electron micrograph of a cortical synapse (postsynapse is to the lower right) after immunogold labeling using anti HA- (black arrowhead, 10 nm gold) and anti-PSD95 antibodies (white arrowhead, 15 nm gold). The HA-NRG1^FL^ transgene preferentially localizes to the PSD95-positive postsynaptic compartment. Scale bar, 100 nm. [Color figure can be viewed in the online issue, which is available at wileyonlinelibrary.com.]

To define the role of BACE1-processed NRG1 type III for myelination *in vivo* and to compare its function with that of full-length NRG1 type III, we generated two transgenic mouse lines that overexpress HA-NRG1^FL^ and HA-NRG1^GIEF^ under control of the neuronal Thy1.2 promoter (Caroni,^1997^) (Fig. 3A). By RT-PCR, both transgenes were expressed already perinatally in brain (data not shown) and spinal cord (Fig. 3B), i.e., before myelination. Western blotting confirmed HA-NRG1^FL^ and HA-NRG1^GIEF^ protein in spinal cord (Fig. 3C), presumably spinal motor neurons (Fig. 3D). Both NRG1 isoforms also accumulated on the surface of large DRG sensory neurons (Fig. 3D). In the brain, transgene expression was prominent in cortical projection neurons, but absent from interneurons and glia cells (Fig. 4A). Interestingly, both HA-NRG1^FL^ and HA-NRG1^GIEF^ preferentially accumulated in neuronal somata and dendrites, including dendritic spines (Fig. 4B right panel), whereas the expected axonal expression was not pronounced (Fig. 4B left panel, arrowheads). Immunoelectron microscopy demonstrated a co-localization of HA-NRG1^FL^ with PSD95 in postsynaptic densities of cortical synapses (Fig. 4C).

**Fig. 5:**
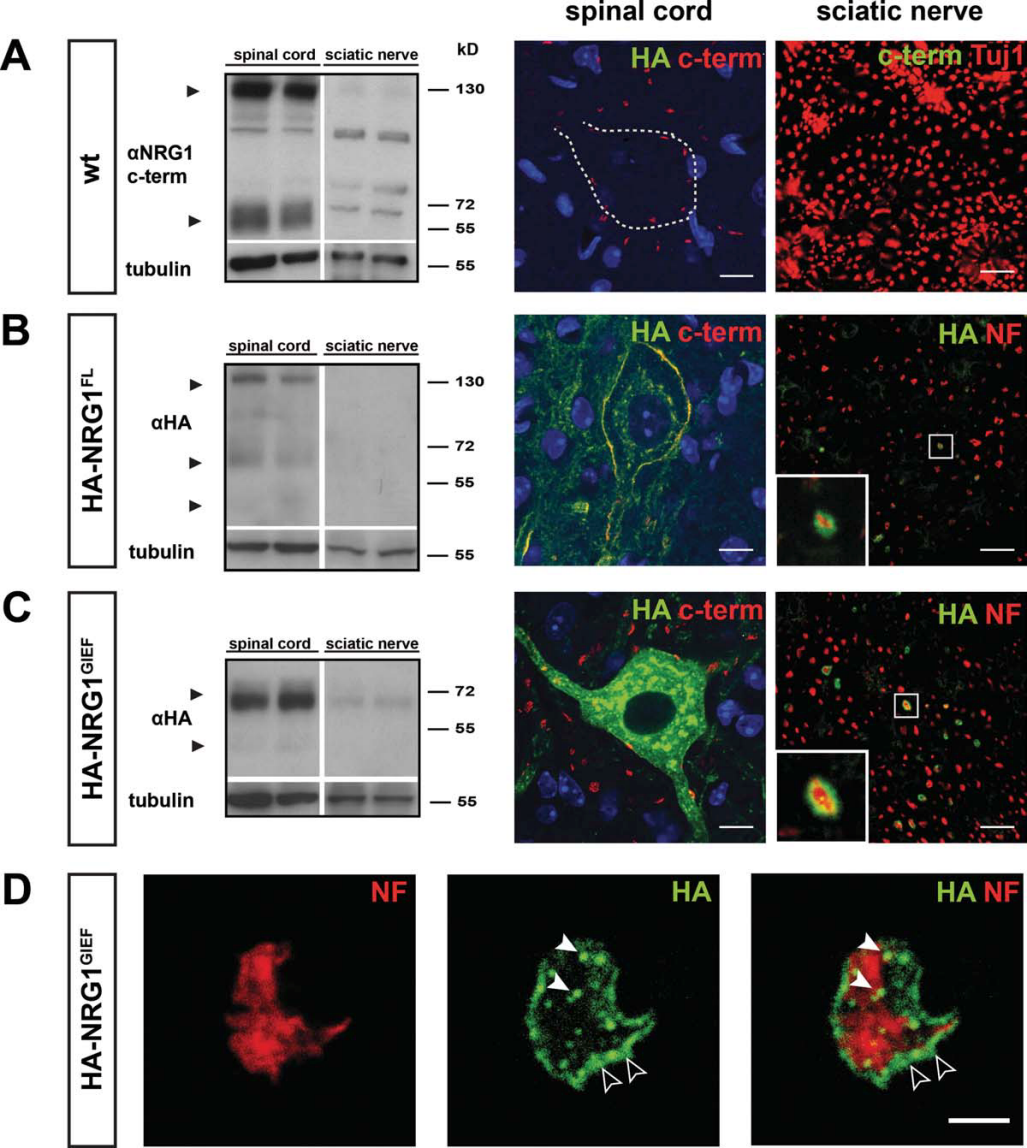
Limited axonal transport of NRG1 type III. (**A**) (left panel) Endogenous full-length NRG1 (140 kD) and a C-terminal processing product (∼ 60 kD; arrowheads) are detected with an antibody against the C-terminus of NRG1 (αNRG1 C-term) in wt spinal cord protein lysates at P12, whereas corresponding sciatic nerve expression is virtually absent. (right panel) Endogenous NRG1 accumulates on the plasma membrane of spinal cord motor neurons after immunostaining with the αNRG1 C-term antibody. Nuclei are counterstained with DAPI (blue). Corresponding NRG1 immunoreactivity is absent from the sciatic nerve. Sciatic nerve axons are marked by tubulin (Tuj1; red). (**B,C**) (left panels) When probed with an anti HA-antibody (αHA), HA-NRG1 type III (140 kD) and two N-terminal processing products (∼ 70 kD and ∼ 45 kD; arrowheads) are present in spinal cord protein lysates from HA-NRG1^FL^ transgenic mice at P12, but barely detectable in sciatic nerve. Similarly, HA-NRG1^GIEF^ (∼ 70 kD) and a smaller protein species (∼ 45 kD; arrowheads) are prominently expressed in spinal cord lysates from HA-NRG1^GIEF^ mice, whereas sciatic nerve expression is low. (right panels) Immunostaining for axons (neurofilament 200, NF; red) and the HA-epitope (green) reveals localization of HA-NRG1^FL^ and HA-NRG1^GIEF^ at the surface of individual sciatic nerve axons (insets). Scale bars, 10 μm. (**D**) Confocal images of an individual sciatic nerve axon in cross section from an adult HA-NRG1^GIEF^ transgenic mouse. Immunostaining for neurofilament 200 (NF; red) and the HA tag (green) reveals expression of HA-NRG1^GIEF^ in vesicle-like structures (white arrowheads) and on the axonal surface (empty arrowheads). Scale bar, 2.5μm. [Color figure can be viewed in the online issue, which is available at wileyonlinelibrary.com.]

The level of neuronal NRG1 type III expression determines myelin sheath thickness of PNS axons (Michailov et al.,^2004^). Thus, we addressed axonal expression of NRG1 type III. We performed Western blots of tissue lysates with an antibody directed against the common C-terminus of NRG1 and probed wildtype mice at P12 and 2 months of age. This readily detected the expression of full-length NRG1 and a proteolytically cleaved C-terminal fragment (CTF) in spinal cord, but these proteins were barely detectable in sciatic nerves (Fig. 5A; Supp. Info. Fig. 2A). Using the same antibody, immunostaining of spinal cord (Fig. 5A) revealed the patchy accumulation of NRG1 on the surface of ventral horn motor neurons, most likely at postsynaptic sites (Issa et al.,^2010^). In contrast, this antibody revealed little NRG1 expression on axonal cross sections of the sciatic nerve (Fig. 5A). Inthese experiments, we used an antibody against Necl1 as a positive control for the detectability of axonal membrane proteins (Supp. Info. Fig. 3A,B).

By probing for the HA epitope, we also detected transgene-derived NRG1 type III in tissue lysates from HA-NRG1^FL^ (Fig. 5B; Supp. Info. Fig. 2B) and HA-NRG1^GIEF^ mice (Fig. 5C; Supp. Info. Fig. 2C). We observed prominent transgene expression in the spinal cord of both lines, but sciatic nerve expression was again low in HA-NRG1^GIEF^ and barely detectable in HA-NRG1^FL^ mice. Similar to the endogenous protein, immunostaining of the HA epitope revealed prominent expression of both transgenes in spinal motor neurons, but only a modest axonal surface expression in a subset of sciatic nerve axons (Fig. 5B,C). Here, we detected the HA-NRG1^GIEF^ variant by confocal microscopy in vesicle-like structures of the axonal lumen, in addition to its surface localization (Fig. 5D). Taken together, these data suggest that axonal delivery of NRG1 type III is severely restricted *in vivo*, and that the steady-state level of mature NRG1 type III on the axonal surface is regulated, at least in part, by limited transport.

### NRG1^GIEF^ Promotes Myelination *In Vivo*

**Fig. 6:**
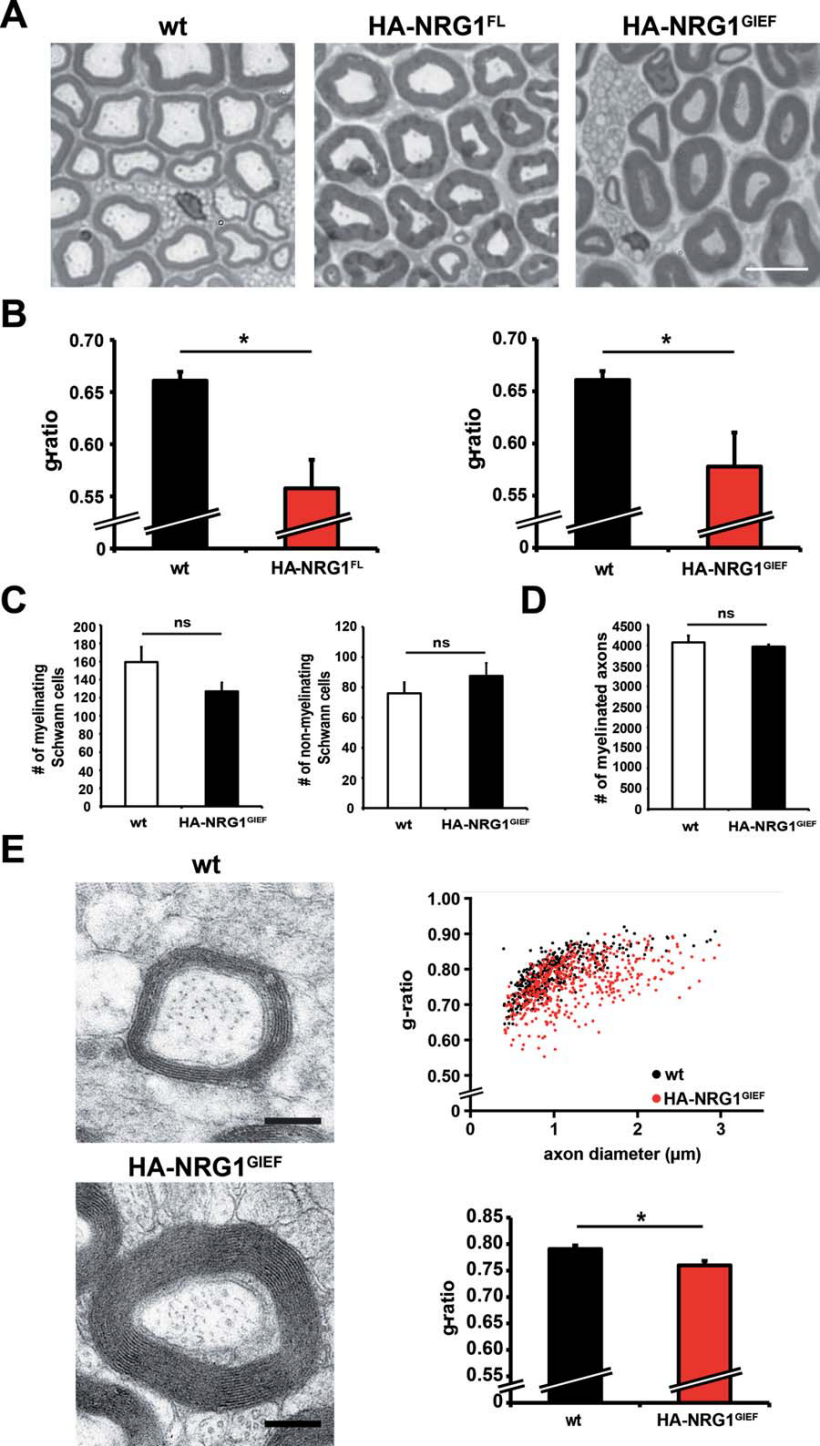
HA-NRG1^GIEF^ promotes hypermyelination in the PNS and CNS of transgenic mice. (**A**) Sciatic nerve hypermyelination in HA-NRG1^FL^ and HA-NRG1^GIEF^ transgenic mice. Methylenblue-Azure II staining for myelin on semithin sciatic nerve cross sections at 2 months of age. Scale bar, 10 μm. (**B**) Quantification of myelin sheath thickness by *g*-ratio analysis. HA-NRG1^FL^ and HA-NRG1^GIEF^ transgenic mice exhibit significantly thicker myelin compared with wt (*P < 0.05). Bars represent mean *g*-ratios ± SEM (*n* = 3; >100 axons/mouse). (**C**) The number of myelinated and nonmyelinated Schwann cells is not changed in HA-NRG1^GIEF^ transgenic mice compared with wt at 2 months of age (*n* = 3; mSchwann cells *P* = 0.08; nmSchwann cells *P* = 0.18). Error bars, ±SEM. (**D**) The total number of myelinated axons in sciatic nerves from HA-NRG1^GIEF^ transgenic mice at 2 months of age is unaltered (*n* = 3 each; *P* = 0.29). Error bars, ±SEM. (**E**) Electron micrographs of the corpus callosum at 2 months of age. Scatter plot (upper panel) displays *g*-ratios as a function of axon diameter. The average *g*-ratio (lower panel) in HA-NRG1^GIEF^ transgenic mice is significantly reduced when compared with wt (wt, *n* = 3; HA-NRG1^GIEF^*n* = 4; *P < 0.05). Error bars, ±SEM. Scale bar, 0.25 μm. [Color figure can be viewed in the online issue, which is available at wileyonlinelibrary.com.]

To assess the capacity of the “preprocessed” GIEF-variant to also promote myelination *in vivo*, we performed a morphological analysis of sciatic nerves at 2 month of age (Fig. 6A). In HA-NRG1^GIEF^ transgenic mice, peripheral myelin was significantly thicker (*g*-ratio: 0.578 ± 0.033) than myelin from age-matched controls (*g*-ratio: 0.661 ± 0.009; *P* < 0.05), which is similar to hypermyelination found in HA-NRG1^FL^ transgenic mice (*g*-ratio: 0.558 ± 0.027; *P* < 0.05) (Fig. 6B).

We note that HA-NRG1^GIEF^ stimulated physiological myelin growth, without myelin pathology except for rare tomaculae (not shown). Furthermore, the number of myelinating (wt 159.33 ± 16.75; HA-NRG1^GIEF^ 127 ± 9.61; *P* = 0.08) and nonmyelinating Schwann cells (wt 76 ± 7.23; HA-NRG1^GIEF^ 87.67 ± 8.41 *P* = 0.18) was not significantly altered in adult HA-NRG1^GIEF^ transgenic mice (Fig. 6C), demonstrating that the GIEF variant is not a potent mitogen for Schwann cells. When we quantified myelinated axons in sciatic nerves of HA-NRG1^GIEF^ transgenic mice, their absolute number was not significantly different from controls (wt 4068.67 ± 164.80; HA-NRG1^GIEF^ 3963.67 ± 49.65; *P* = 0.29) (Fig. 6D), demonstrating that chronic HA-NRG1^GIEF^ overexpression has no adverse effects on axonal integrity.

We have previously reported that neuronal overexpression of full-length NRG1 type III causes hypermyelination in the CNS (Brinkmann et al.,^2008^). To determine if oligodendrocytes respond to neuronally overexpressed HA-NRG1^GIEF^, we analyzed *g*-ratios in the corpus callosum of HA-NRG1^GIEF^ transgenic mice by electron microscopy. At 2 months of age, callosal fibers were significantly hypermyelinated (0.760 ± 0.009) compared with wildtype (0.790 ± 0.007; *P* < 0.05) (Fig. 6E), similar in extent to mice overexpressing full-length HA-NRG1^FL^ (not shown).

Collectively, these data demonstrate that “preprocessed” HA-NRG1^GIEF^ possesses all the features required for axonal targeting of the protein and for stimulating Schwann cells and oligodendrocytes to synthesize myelin.

### Is Proteolytic Processing Required for NRG1 Type III Signaling Activity?

**Fig. 7:**
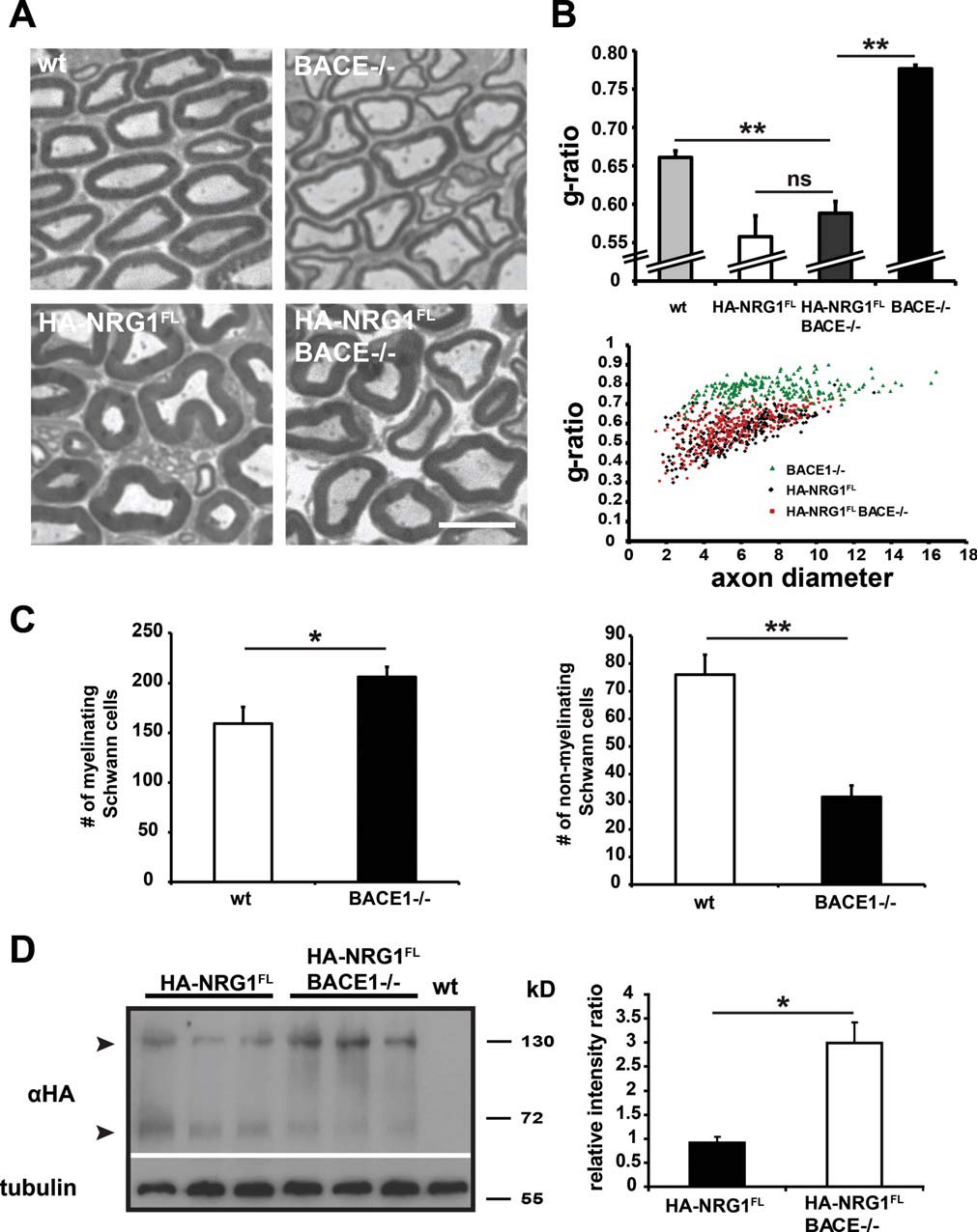
The myelination-promoting competence of NRG1 type III is not compromised in the absence of BACE1. (**A**) Semithin sciatic nerve cross sections stained for myelin at 2 months of age. Sciatic nerves from BACE1−/− mutants are severely hypomyelinated and HA-NRG1^FL^ transgenic nerves are hypermyelinated compared with wt. Expression of the HA-NRG1^FL^ transgene in a BACE1 null background restores myelination. Scale bar, 10 μm. (**B**) Quantification of myelin sheath thickness by g-ratio analysis. HA-NRG1^FL^ overexpression in BACE1−/− mutants significantly increases myelin sheath thickness compared with BACE1−/− mutants (**P < 0.01) and even wt (**P < 0.01). Myelin sheath thickness in HA-NRG1^FL^*BACE1−/− mutants is not significantly reduced compared with HA-NRG1^FL^ transgenic mice (*P* = 0.35). (*n* = 3 per genotype, >100 axons/mouse). The scatter plot (lower panel) illustrates that HA-NRG1^FL^ expression in BACE1−/− mice rescues the myelination deficit of BACE1 mutants across all axon diameters. (**C**) Myelinating Schwann cells (left panel) are increased and nonmyelinating Schwann cells (right panel) decreased in adult BACE1−/− mutants (*n* = 4) compared with wt (*n* = 3) at 2 months of age (mean±SEM; *P < 0.05; **P < 0.01) (**D**) “Stalk” cleavage of NRG1 type III is reduced but not abolished in the absence of BACE1. Western blot of spinal cord protein lysates from 2 months old mice probed with an anti-HA antibody. Processed NRG1 type III (∼70 kD) is reduced, whereas full-length NRG1 type III (∼140 kD) accumulates in HA-NRG1^FL^*BACE1−/− compound mutants compared with HA-NRG1^FL^ transgenic mice. Membrane was reprobed for tubulin as a loading control. Densitometric quantification demonstrates a significant increase in the relative signal ratio (∼140 kD/∼70 kD protein bands relative to tubulin) in HA-NRG1^FL^*BACE1−/− mice ± SEM; *P < 0.05). [Color figure can be viewed in the online issue, which is available at wileyonlinelibrary.com.]

To determine whether NRG1 signaling depends on BACE1 activity, we analyzed transgenic mice with elevated levels of full-length NRG1 type III in combination with complete BACE1 deficiency. We confirmed that sciatic nerves are hypomyelinated in BACE1 null mutants (wt 0.661 ± 0.009; BACE1−/− 0.776 ± 0.005; *P* < 0.01) (Fig. 7A,B), but also challenged the system by additional NRG1 type III overexpression. Remarkably, sciatic nerves from BACE1 null mutants expressing full-length transgenic mice after HA-NRG1^FL^ were clearly hypermyelinated (0.588 ± 0.015; *P* < 0.01) (Fig. 7A,B). Thus, BACE1 deficiency did not reduce the ability of HA-NRG1^FL^ to promote myelination as evidenced by *g*-ratios that were not significantly different in NRG1^FL^*BACE1−/− mutants and HA-NRG1^FL^ transgenic mice (0.558 ± 0.027; *P* = 0.35) (Fig. 7A,B).

In addition to myelination, NRG1 signaling is critically important for the expansion and survival of the Schwann cell precursor pool during embryonic development (Meyer and Birchmeier,^1995^; Wolpowitz et al.,^2000^). Thus, we addressed whether the loss of BACE1-mediated processing of NRG1 affects Schwann cell numbers. When quantified at 2 months of age, the number of myelinating Schwann cells in BACE1 mutants was increased compared with wildtype (wt 159.33 ± 16.75; BACE1−/− 206.25 ±1 0.21; *P* < 0.05), whereas nonmyelinating Schwann cells were concomitantly decreased (wt 76 ± 7.23; Bace1−/− 31.75 ± 4.15; *P* < 0.01) (Fig. 7C), such that the total number of Schwann cells was unchanged. These data strongly suggest that BACE1 is not critically required for the initial expansion and survival of the Schwann cell precursor pool.

Finally, we monitored the processing of HA-NRG1^FL^ in a BACE1 null mutant background. In the spinal cord of HA-NRG1^FL^ transgenics, the relative ratio of full-length/processed NRG1 type III was 0.92 ± 0.13 (Fig. 7D). On the BACE1-null background, we found an increased ratio, i.e., accumulation of full-length NRG1 type III and reduction of the processed isoform (2.99 ± 0.44; *P* < 0.05). However, also BACE1 null mutants harbor about 50% processed transgene-derived NRG1 type III in the spinal cord, but the responsible protease has not been identified (Fig. 7D).

## DISCUSSION

We have used novel NRG1 transgenic mouse lines to directly prove the hypothesis that BACE1-mediated cleavage of NRG1 type III produces an axonal myelination signal (Willem et al.,^2006^). Moreover, we confirm the hypothesis that the juxtamembrane stalk region of full-length NRG1 type III is the critical site for BACE1 processing *in vivo*. Transgenic expression of a “preprocessed” NRG1 type III isoform (NRG1^GIEF^) promotes myelination *in vivo* and rescues the myelination defect when NRG1 type III-deficient sensory neurons are co-cultured with myelination competent Schwann cells. Thus, NRG1^GIEF^ provides all necessary protein functions to stimulate the myelination program of Schwann cells, implying that the C-terminal domain of NRG1 type III is dispensable.

Interestingly, in the absence of BACE1, we found proteolytic cleavage of the NRG1 type III stalk region to be reduced but not completely abolished. Moreover, breeding BACE1-deficient mice to NRG1 type III transgenic mice rescued peripheral hypomyelination and even produced hypermyelination. Also, normal numbers of Schwann cells in BACE1 mutants demonstrate that the NRG1 type III dependent expansion of the Schwann cell precursor pool is not markedly compromised. These data suggest that either additional proteases participate in the processing of NRG1 type III or that full-length NRG1 type III has sufficient signaling activity to promote the initial stages of Schwann cell development and myelination.

### Proteolytic Cleavage in the Stalk Region Produces a Myelination-Inducing NRG1 Type III Variant

NRG1^GIEF^ restores myelination of NRG1 type III-deficient sensory neurons and promotes myelination in the PNS and CNS of transgenic mice despite lacking the “classic” transmembrane domain and the C-terminal cytoplasmic tail. Thus, these protein domains are not essential for correct axonal presentation by NRG1 type III and for stimulation of glial ErbB receptors. Moreover, neuronal “back signaling” of the CTF after cleavage (Bao et al.^2003^; Bao et al.,^2004^) appears dispensable for myelination.

NRG1^GIEF^ (derived from NRG1 type IIIβ1a) closely resembles the “sensory and motor neuron derived factor” SMDF (NRG1 type IIIβ3), a natural splice variant of NRG1 type III (Ho et al.,^1995^). In contrast to NRG1^GIEF^, neuronal overexpression of SMDF in transgenic mice did not induce hypermyelination but potently stimulated proliferation of nonmyelinating Schwann cells, resulting in neurofibroma-like neoplasia (Gomez-Sanchez et al.,^2009^). This functional heterogeneity between NRG1^GIEF^ and SMDF most likely resides in the distinct C-terminus of SMDF, which may serve as an acylation-like post-translational modification site (Cabedo et al.,^2002^), thereby resulting in a tighter membrane-association of SMDF (including its EGF-like domain) when compared with NRG1^GIEF^. Thus, SMDF may differentially stimulate glial ErbB receptors and downstream signaling pathways.

Similar to SMDF, transgenic overexpression of a soluble NRG1 type IIβ3 variant (glial growth factor; GGF) in myelinating Schwann cells causes glial hyperplasia, demyelination, and onion bulb formation (Huijbregts et al.,^2003^). Thus, chronic exposure to several NRG1β3 variants can exert adverse effects on nervous system integrity *in vivo*. In contrast, we show here that HA-NRG1^GIEF^ (the β1a isoform) is a potent stimulator of myelination without affecting Schwann cell numbers or inducing major myelin abnormalities.

### Limited Axonal Transport of NRG1 Type III

Because of the lack of suitable antibodies, the subcellular localization of the N-terminal portion of NRG1 in the nervous system has been difficult to address. Thus, our transgenic mice that express epitope-tagged NRG1 type III variants represent novel tools to study the expression, processing, and subcellular targeting of NRG1 type III *in vivo*. We demonstrate axonal surface localization of the N-terminal domain of NRG1 type III and observe luminal expression of processed NRG1 type III, indicating axonal targeting via vesicular transport. Moreover, our data suggest that axonal transport may be rate limiting for myelination, providing a potential mechanism how the level of axonal NRG1 type III is tightly regulated during myelination.

Several mechanisms dictate the differential distribution of membrane proteins in neurons, including polarized delivery into the dendritic compartment followed by internalization, transcytosis, and axonal targeting (Winckler and Mellman,^2010^). As NRG1 type III is prominently targeted to the somatodendritic compartment (Bare et al.,^2011^), we suggest that axonal transport of NRG1 type III follows such an indirect pathway.

### NRG1 Type III Serves as a Myelination Signal in the Absence of BACE1 Processing

Hypomyelination of peripheral nerves in BACE1 mutants has been linked to impaired processing and activation of NRG1 type III (Willem et al.,^2006^). We provide direct evidence that BACE1 cleavage occurs in the stalk region of full-length NRG1 type III, and that BACE1-processed NRG1 type III (NRG1^GIEF^) is a myelination signal. However, our findings suggest that NRG1^GIEF^ represents only a fraction of all N-terminal NRG1 fragments that we detect in transgenic mice when probing for the N-terminal HA epitope. We conclude that cleavage of NRG1 type III by BACE1 and other proteases produces several myelination-inducing N-terminal NRG1 fragments with similar size but slightly different C-termini. The activity of additional proteases is further supported by our genetic gain-of-function experiments that have identified an unexpected independence of NRG1 type III signaling from BACE1 activity during myelination. Stalk cleavage of NRG1 type III is only modestly reduced in the absence of BACE1, and simple breeding to NRG1 type III transgenic mice fully restores myelination in BACE1 null mutants. This demonstrates that NRG1 type III is also functionally active in the absence of BACE1. Functional NRG1 signaling in the absence of BACE1 is further supported by normal number of Schwann cells in sciatic nerves from BACE1 null mice.

We conclude that BACE1-mediated activation of NRG1 is not required for the initial expansion and survival of the Schwann cell precursor pool during embryonic development, a process that critically depends on NRG1 (Meyer and Birchmeier,^1995^; Wolpowitz et al.,^2000^). Although the total number of Schwann cells is unchanged, we observed an increase in myelinating Schwann cells and a concomitant reduction of nonmyelinating Schwann cells in BACE1 deficient mice. Thus, BACE1 may regulate Schwann cell fate. Whether this involves NRG1 processing (or other targets) requires further investigation.

Disruption of NRG1 in motor neurons during adulthood impairs axonal regeneration and remyelination after nerve injury (Fricker et al.,^2011^). Although remyelination after nerve injury was also reduced in BACE1 null mutants (Hu et al.,^2008^), axonal regeneration was enhanced (Farah et al.,^2011^), further supporting the hypothesis that NRG1 signaling in the PNS is, at least in part, independent from BACE1.

This unexpected independence of NRG1 type III signaling from BACE1 function could be explained either by sufficient signaling activity of full-length NRG1 type III or by the activity of additional proteases that also process NRG1 type III (or by a combination of both). Noncleavable variants of TGF-beta can activate the EGF (ErbB1) receptor (Brachmann et al.,^1989^; Wong et al.,^1989^) and plasma membrane preparations harboring human NRG1 type I stimulate ErbB2 phoshorylation and DNA synthesis in cultured cells (Aguilar and Slamon,^2001^). This supports the idea that also full-length ligands for ErbB receptors are functionally active.

Compensating for BACE1 could be ADAMs metalloproteases that were shown to process NRG1 type I-variants *in vitro*, including ADAM8 (Guaiquil et al.,^2010^), ADAM10 (Luo et al.,^2011^), ADAM17/TACE (Horiuchi et al.,^2005^; Montero et al.,^2000^), and ADAM19/β-meltrin (Shirakabe et al.,^2001^; Yokozeki et al.,^2007^). Because TACE has been identified as a negative regulator of myelination (La Marca et al.,^2011^), it may inactivate rather than activate NRG1. Similarly, pharmacological inactivation of ADAMs proteases (our study) or matrix metalloproteinase-28 (Werner et al.,^2008^) stimulates myelination *in vitro*. Therefore, the exact function of other ADAMs metalloproteinases in myelination and their role in NRG1 type III processing (including shedding of the EGF-like domain) will be an important subject of future research.

## Supplementary material

Supporting Information Figure 1. In vitro myelination proceeds in the absence of neuronal BACE1 (A) Immunostaining for MBP (red) and NF (blue) of DRG neuron-Schwann cell co-cultures after 10 and 13 days in myelinating conditions. Cultures with BACE1-/- DRG neurons are myelinated with considerably less MBP positive myelin segments compared to wt cultures. Scale bar, 100 μm. (B) Western blots of DRG neuron-Schwann cell co-cultures. Cultures with BACE1-/- neurons have less MBP compared to wt in line with the decreased number of myelin segments. Membrane was reprobed for actin as a loading control.

Supporting Information Figure 2. Limited axonal transport of NRG1 type III in mature sciatic nerve (A-C) Western blots of spinal cord and sciatic nerve protein lysates (40 μg) from mice at 2 months of age. Membranes were reprobed for tubulin as a loading control. (A) Using an antibody directed against the C-terminus of NRG1 (αNRG1 C-term), endogenous full length NRG1 (∼140 kD) and a C-terminal processing product (∼60 kD; arrowheads) are detected in wt spinal cord, whereas corresponding sciatic nerve expression is virtually absent. Asterisks mark unspecific bands. (B) When probed with an anti HA-antibody (αHA), full length HA-NRG1 type III (140 kD) and two N-terminal processing products (∼70 kD and ∼45 kD; arrowheads) are expressed in spinal cord, but not sciatic nerve lysates from HA-NRG1FL transgenic mice. (C) HA-NRG1GIEF (∼70 kD) as well as a smaller protein species (∼45 kD; arrowheads) are prominently expressed in spinal cord lysates from HA-NRG1GIEF mice, whereas the corresponding expression in sciatic nerve is low.

Supporting Information Figure 3. Axonal membrane protein Necl1 is expressed in the mature sciatic nerve (A) Western blotting reveals Necl1 expression (48 kD) in spinal cord and sciatic nerve protein lysates prepared from wt mice at two moth of age (same lysates as in Suppl. Fig. 2A). (B) Necl1 is expressed on the surface of sciatic nerve axons in adult mice. Fluorescent immunostaining of a sciatic nerve cross section derived from a HA-NRG1FL transgenic mouse (age four months) stained for Necl1 (red) and tubulin (Tuj1; green). Scale bar, 10 μm.
