# Supplementary figures and images for: BACE1 Processing of NRG1 Type III Produces a Myelin-Inducing Signal but Is Not Essential for the Stimulation of Myelination
Source: Glia. 2011 Nov 2;60(2):203–17. doi: 10.1002/glia.21255 (PMC3267053; doi:10.1002/glia.21255)

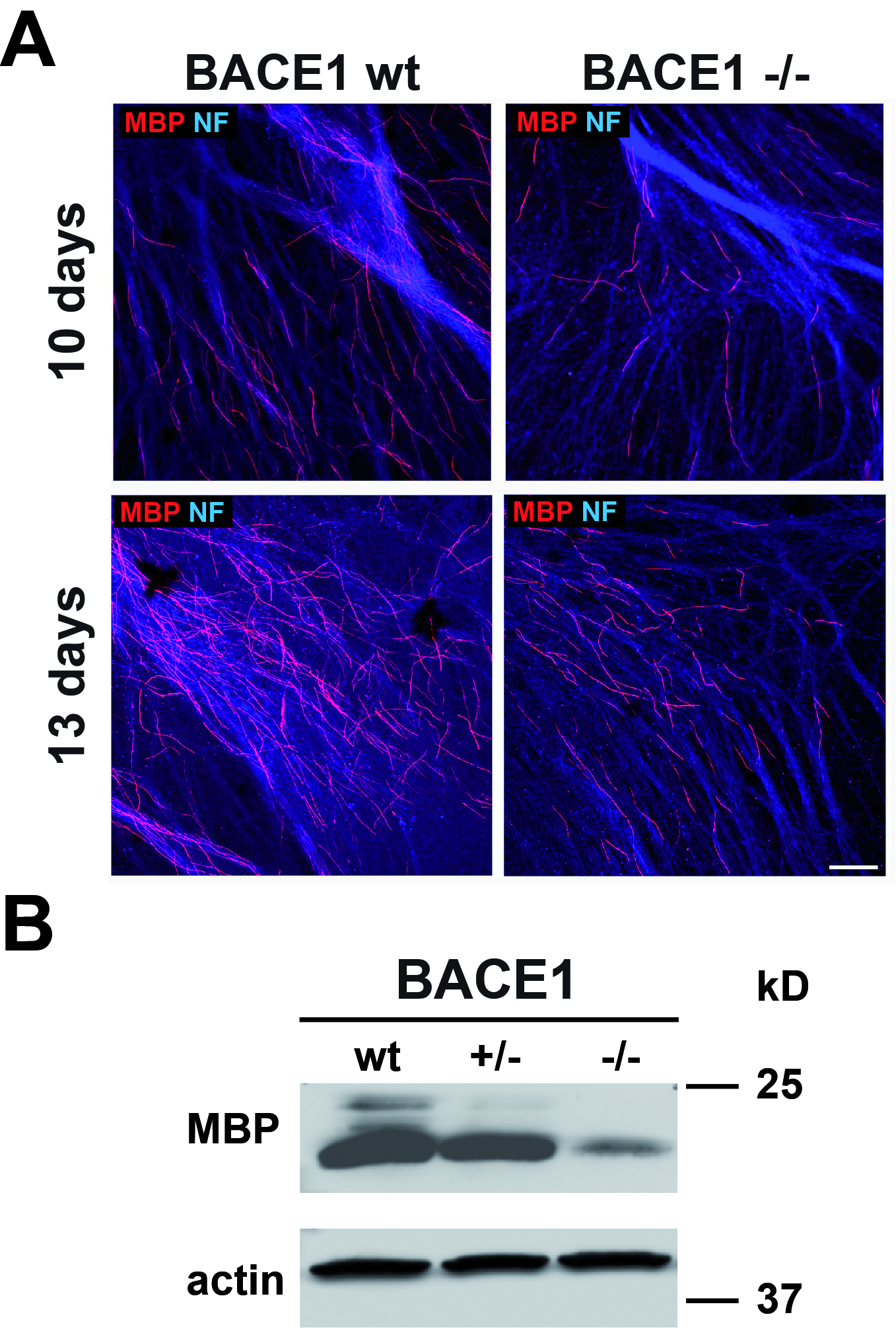

Supplement: Supplementary file 1 [file glia0060-0203-SD1.tif]

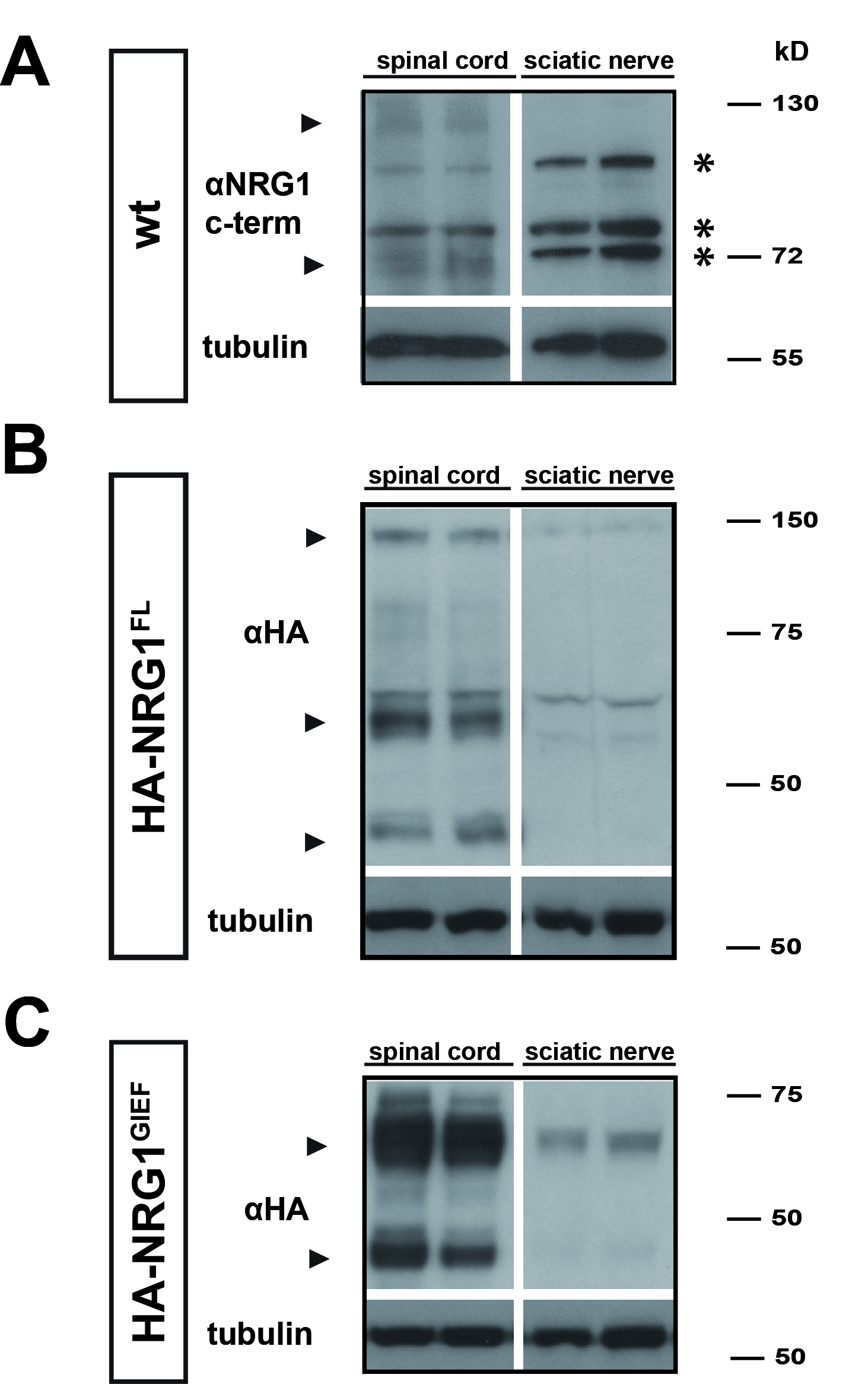

Supplement: Supplementary file 2 [file glia0060-0203-SD2.tif]

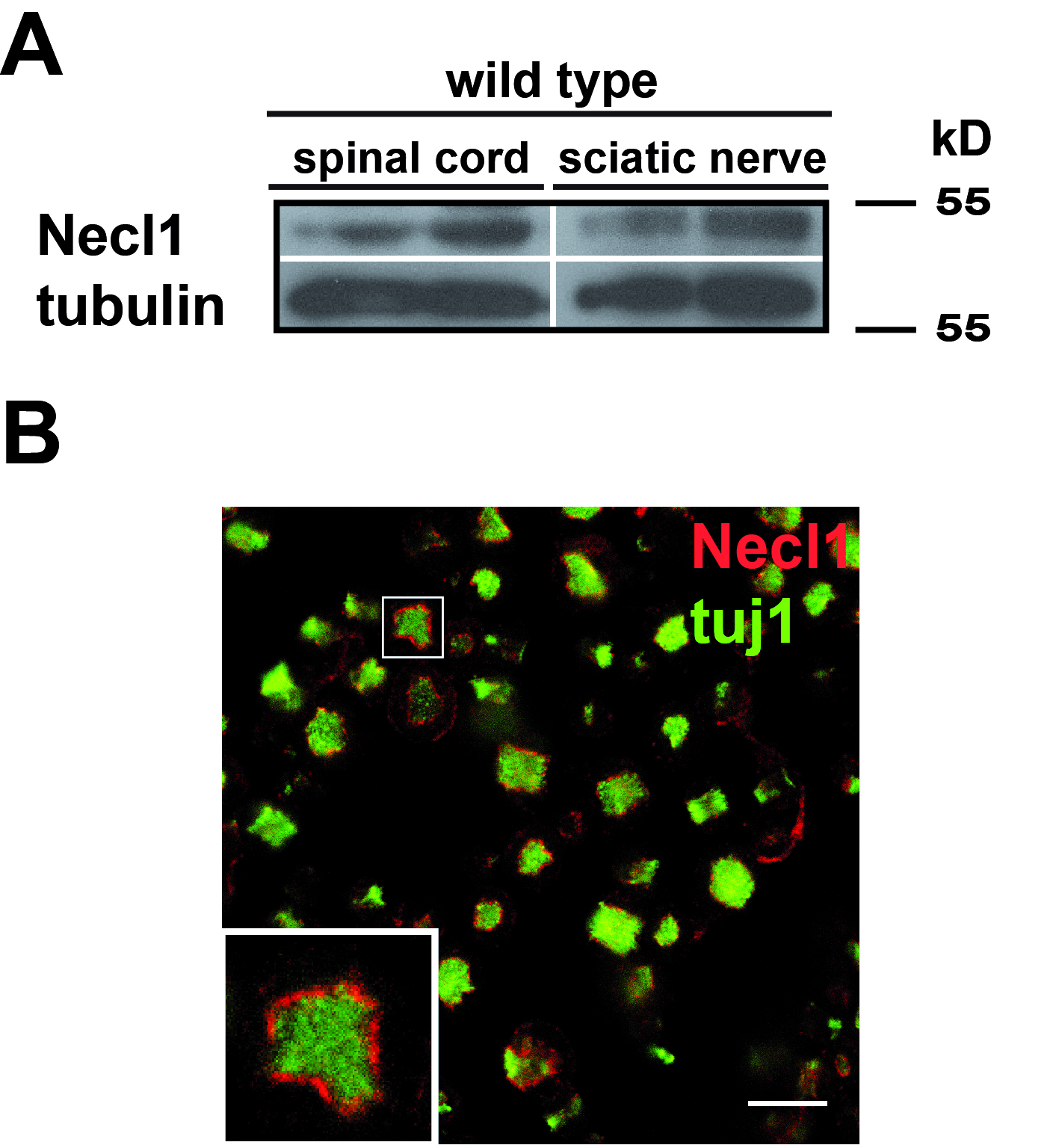

Supplement: Supplementary file 3 [file glia0060-0203-SD3.tif]
